# Ginsenoside Rg3 inhibits the senescence of prostate stromal cells through down-regulation of interleukin 8 expression

**DOI:** 10.18632/oncotarget.17616

**Published:** 2017-05-04

**Authors:** Yanfei Peng, Ran Zhang, Lingfei Kong, Yongmei Shen, Da Xu, Fang Zheng, Jianwei Liu, Qian Wu, Bona Jia, Ju Zhang

**Affiliations:** ^1^ School of Integrative Medicine, Tianjin University of Traditional Chinese Medicine, Tianjin, China; ^2^ Department of Biochemistry and Molecular Biology, College of Life Science, Bioactive Materials Key Lab of Ministry of Education, Nankai University, Tianjin, China; ^3^ Department of Pharmaceutics, School of Pharmacy, Rutgers University, New Brunswick, New Jersey, USA

**Keywords:** ginsenoside Rg3, IL-8, prostate cancer, senescence, stromal cell, Gerotarget

## Abstract

Senescent stromal cells support the development of prostate cancer and are considered potential therapeutic targets. This research evaluated the regulatory effects of ginsenoside Rg3 on the senescence of prostatic stromal cells pre-incubated in medium supplemented with 0.5% fetal bovine serum. The results revealed that ginsenoside Rg3 decreased the number of stromal cells positively stained with a senescent cell marker (senescence-associated β-galactosidase). Ginsenoside Rg3 also increased the viability of stromal cells and promoted cell cycle transition from G0/G1 to S phase, as well as inhibited the carcinoma-associated fibroblast-like phenotype in prostate stromal cells, through the up-regulation of smooth muscle cell markers SM22 and smooth muscle myosin heavy chain. Conditioned medium collected from stromal cells treated with ginsenoside Rg3 exhibited an attenuated effect on the promotion of prostate cancer cell migration compared with conditioned medium from stromal cells without Rg3 treatment. Down-regulation of interleukin 8 (IL-8) in a dose- and time-dependent manner was observed in ginsenoside Rg3-treated stromal cells, and over-expression or addition of IL-8 reversed the anti-senescence role of Rg3 in prostate stromal cells. Furthermore, ginsenoside Rg3 down-regulated IL-8 expression by decreasing the reactive oxygen species level in prostatic stromal cells and reducing the transcriptional activity of IL-8 promoter by damping the transcription factors C/EBP β and p65 binding to IL-8 promoter. Our research revealed that ginsenoside Rg3 was able to inhibit prostate stromal cell senescence by down-regulating IL-8 expression. The results suggest a potential value for ginsenoside Rg3 in prostate cancer treatment through the targeting of pro-carcinogenic senescent stromal cells.

## INTRODUCTION

Senescence is a specific cellular status under a variety of stimuli with stable cell cycle arrest. Cellular senescence can be classified into replicative and premature. Replicative senescence, induced by telomere shortening during DNA replication, exists in most mammal somatic cells and is presumed to contribute to organismal aging in later adult life, while premature cellular senescence is usually induced by DNA damage, activation of oncogenic signaling, nutrition deprivation or reactive oxygen species (ROS) accumulation. Recent research compared gene expression profiles between replicative senescent cells and cells arrested by DNA damage or serum starvation and revealed that only a small subset of genes were exclusively regulated in replicative senescent cells while the majority of expression changes in the three conditions were similar [1].

Cellular senescence may prevent the malignant transformation of cells through the avoidance of excessive proliferation-accumulated DNA mutations. However, in some heterogeneous tissues such as prostate, senescent stromal cells effectively support the progression of epithelium-derived cancer and are postulated as novel therapeutic targets in cancer treatment [2]. Isolated stromal fibroblasts from elderly men (63 to 81 years old), but not from young men (40 to 52 years old), have been reported to promote the proliferation of prostate epithelial cells by highly expressing CXCL12 [3]. Other research has reported the up-regulation of growth factors and chemokines in senescent prostate stromal cells, inducing the proliferation of cancer cells in a paracrine manner [4]. Recent research indicated that senescent stromal cells supported cancer cell proliferation by secreting energy-rich compounds, induced epithelial-to-mesenchymal transition progression, and subsequently promoted cancer aggressiveness, as well as contributed to the immune escape of cancer cells [5]. Moreover, senescent stromal cells also enhanced the chemoresistance of cancer cells in a paracrine manner [6]. These reports suggest senescent stroma is a potential therapeutic target in prostate cancer treatment.

Ginsenoside Rg3, extracted from a traditional medical herb, ginseng, has been reported as a relatively safe drug with anticancer activity both *in vitro* and *in vivo* [7, 8]. Previous studies evaluating the effects of ginsenoside Rg3 on prostate cancer cells have revealed that the compound inhibited the proliferation and migration of cancer cells, as well as enhanced the susceptibility of cancer cells to chemotherapeutic drugs by inhibiting the NF-κB signaling pathway [9-11]. The beneficial effects of ginsenoside Rg3 on prostate cancer cells have been well recognized, while the impact of this compound on stromal cells is still ambiguous.

In this study, we report the anti-senescence role of ginsenoside Rg3 in prostate stromal cells pre-incubated in medium with low serum concentration. Our results suggest that ginsenoside Rg3 also regulates the phenotype of stromal cells and blocks the promoting effects of stromal cells on prostate cancer cell migration. Furthermore, decrease of IL-8 expression was observed in ginsenoside Rg3-treated stromal cells and down-regulation of IL-8 was essential for the anti-senescence role of ginsenoside Rg3. The research also uncovered that ginsenoside Rg3 attenuated IL-8 expression by damping ROS level and transcriptional activity of IL-8 gene promoter. In conclusion, our results suggested potential beneficial effects of ginsenoside Rg3 in prostate cancer treatment, by the targeting of the stromal component, especially in the senescent stromal environment.

## RESULTS

### Ginsenoside Rg3 prevented the senescence of prostate stromal cells *in vitro*

**Figure 1:**
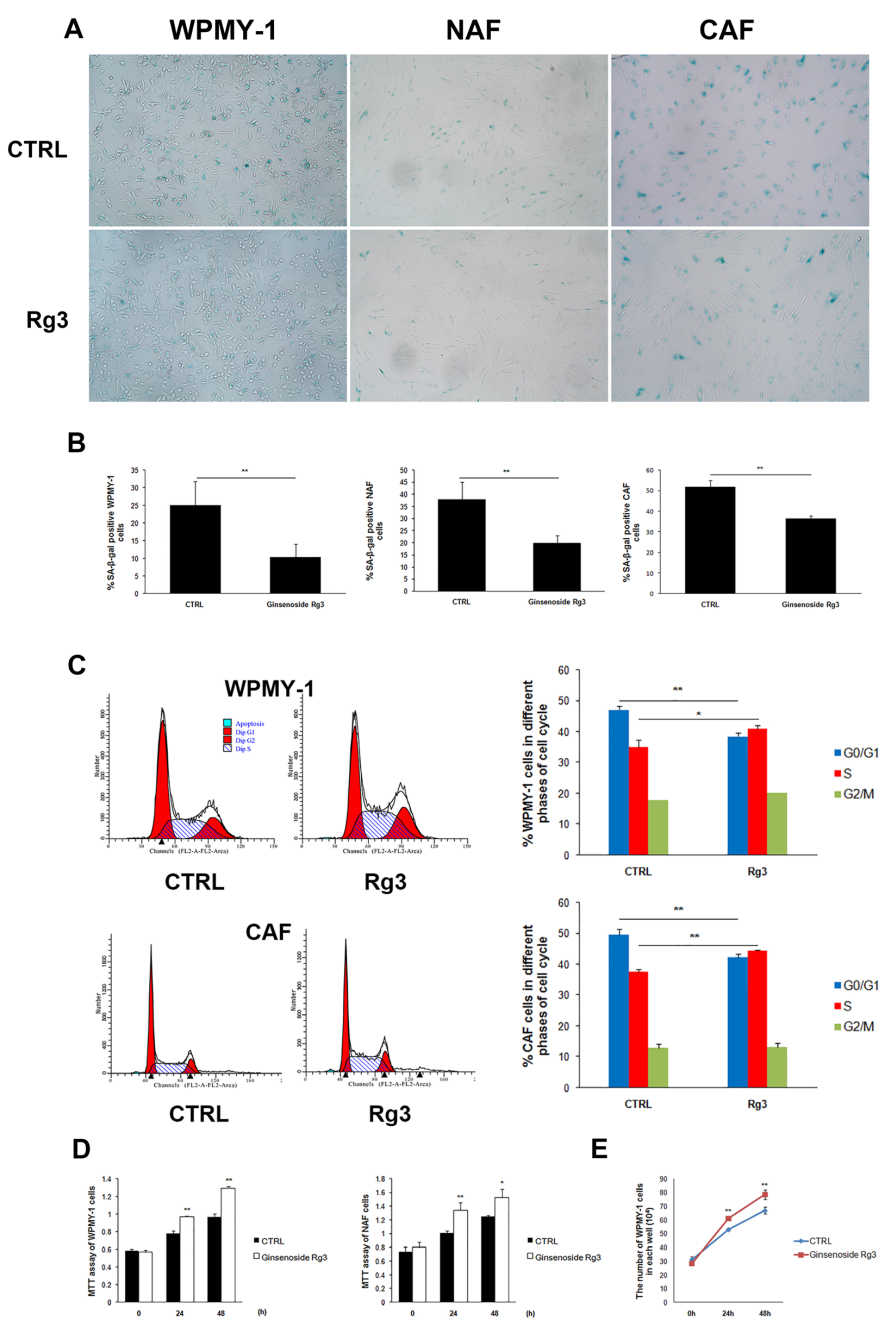
Ginsenoside Rg3 inhibited senescence of prostate stromal cells incubated in serum starvation condition **A.** Images showing SA-β-gal staining in WPMY-1, NAF, and CAF cells. **B.** Quantitative analysis of the percentage of SA-β-gal positive stained cells. **C.** Flow cytometry assay results indicating that Rg3 promoted the cell cycle from G0/G1 to S phase in WPMY-1 and CAF cells. **D.** MTT assay results revealing that ginsenoside Rg3 enhanced the viability of WPMY-1 and NAF cells. **E.** Graph demonstrating that ginsenoside Rg3 increased the proliferation of WPMY-1 cells. Images of WPMY-1 and NAF were collected at ×100 magnification. Images of CAF were at ×200 magnification. The results were obtained from 3 independent experiments and are presented as means ± SD. *p < 0.05; **p < 0.01.

Senescence-associated β-galactosidase (SA-β-gal) is commonly used as a marker of cellular senescence. In this study, normal prostatic stromal cells WPMY-1, NAF, and carcinoma-associated fibroblasts (CAF) cells were cultured in DMEM medium supplemented with 0.5% fetal bovine serum (FBS) for 36 h and subsequently treated with vehicle (DMSO) or 25 μM ginsenoside Rg3 for another 48 h. In all 3 cell lines, cell percentages of SA-β-gal positive staining were significantly down-regulated by ginsenoside Rg3 treatment (Figure 1A and 1B). Cell cycle arrest was also evaluated by flow cytometry analysis and the results showed that ginsenoside Rg3 promoted the transition of the cell cycle from G0/G1 to S phase in WPMY-1 normal stromal cells and CAF carcinoma-associated stromal cells (Figure 1C). Not only was the viability of WPMY-1 and NAF cells enhanced by ginsenoside Rg3, but also the proliferation of WPMY-1 (Figure 1D and 1E). We also studied the effects of ginsenoside Rg3 on primary cultured rat prostate stromal cells and found that the compound not only inhibited the senescence of serum-starved cells but also delayed replicative senescence (Supplementary Figure 1). All these results indicated that ginsenoside Rg3 is an anti-senescent compound in prostate stromal cells.

### Ginsenoside Rg3 up-regulated the expression of the smooth muscle cell markers SM22 and smooth muscle myosin heavy chain (SMMHC) in WPMY-1 and NAF cells

**Figure 2:**
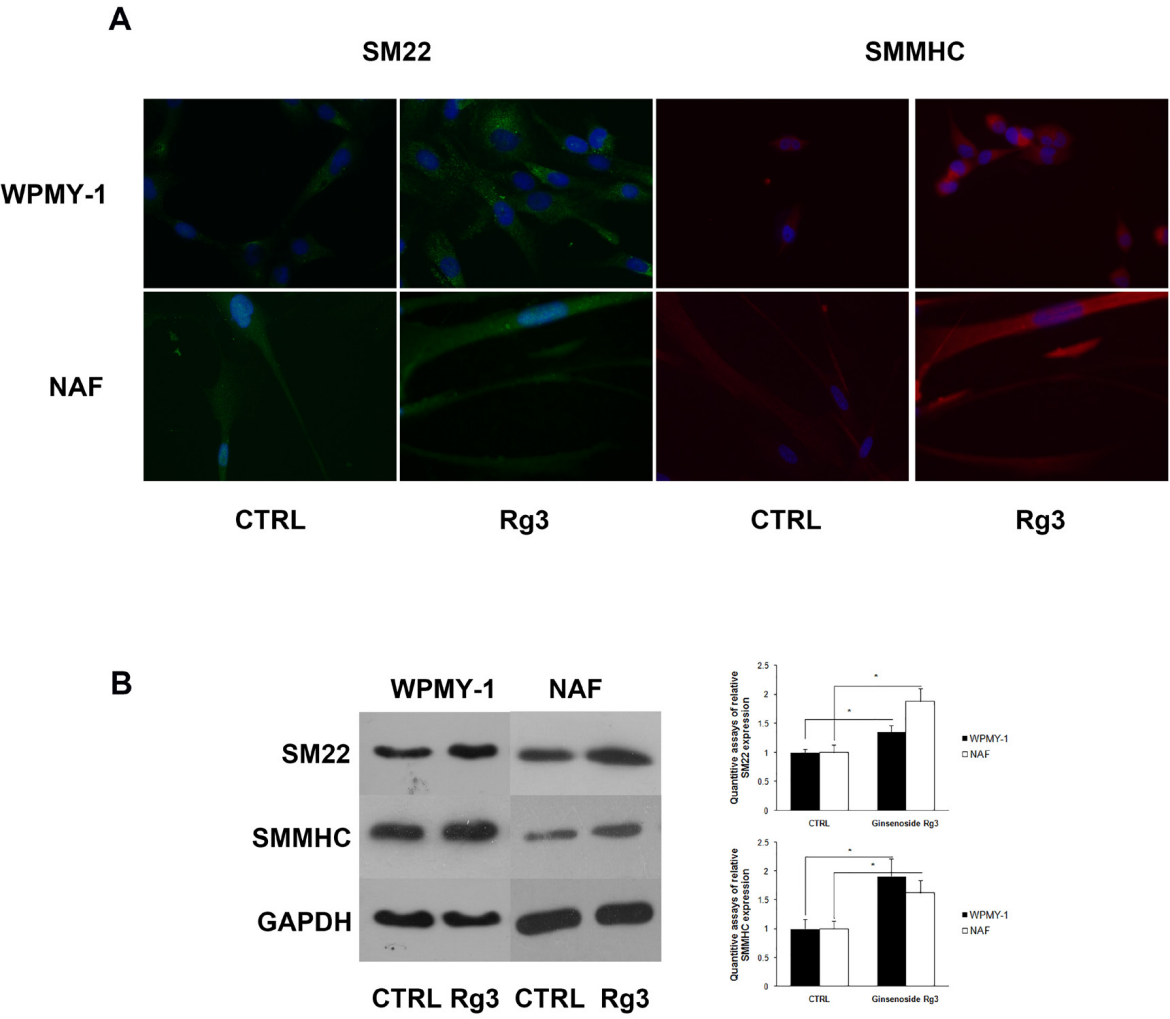
Ginsenoside Rg3 increased SM22 and SMMHC expression in WPMY-1 and NAF cells **A.** Immunofluorescence assays indicating the up-regulation of SM22 (green) and SMMHC (red) induced by ginsenoside Rg3 in WPMY-1 and NAF cells. Blue fluorescence indicates the nucleus stained by DAPI. The images were collected at ×400 magnification. **B.** Western blot assays verifying the results from immunofluorescence. The results were also quantified with Image J software and are presented as means ± SD. *p < 0.05.

The heterogeneity of prostate stromal cells has been widely characterized [12]. The cells mainly comprise fibroblasts and smooth muscle cells with diverse phenotypes. The phenotypic variation of prostate stromal cells has been observed in benign prostatic hyperplasia (BPH) and prostate cancer. In this research, immunofluorescence assays were performed to analyze phenotypic markers in WPMY-1 and NAF (Figure 2A), revealing that ginsenoside Rg3 up-regulated the expression of smooth muscle cell markers SM22 and SMMHC. The same results were obtained by western blot assay (Figure 2B).

### Ginsenoside Rg3 blocked the promoting effects of WPMY-1 on cancer cell migration

**Figure 3:**
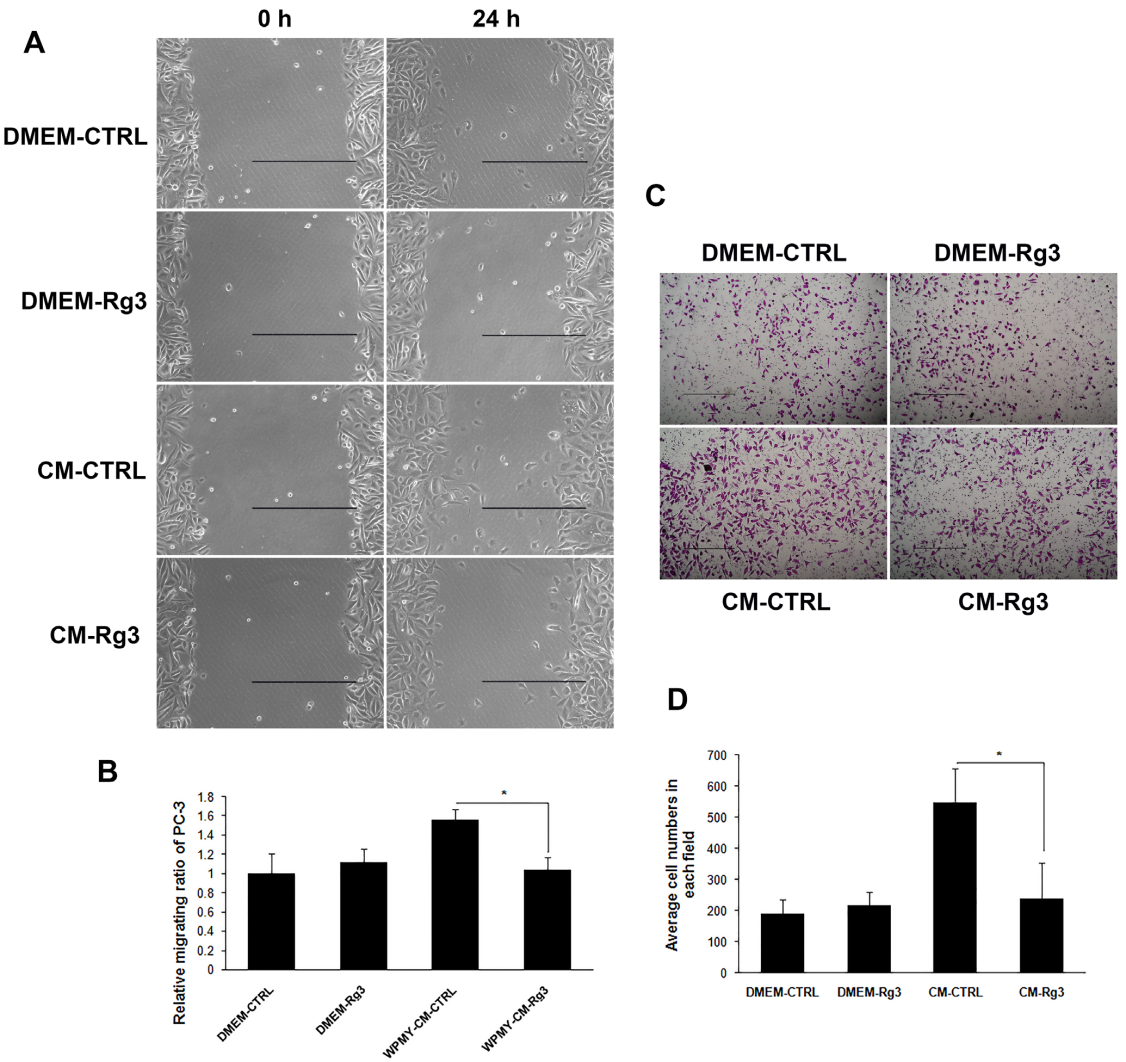
Ginsenoside Rg3 inhibited PC3 cell migration through the modulation of WPMY-1 cell paracrine **A.** Wound healing assays of PC3 cells at 0 and 24 h. The images were collected at ×200 magnification. Scale bar, 500 μm. **B.** Quantification of the results from wound healing assays. **C.** Transwell assays of PC3 cells at 0 and 24 h. The pictures were captured at ×100 magnification. Scale bar, 500 μm. **D.** Quantification of the results from transwell assays. The results were obtained from 3 independent experiments and are presented as means ± SD. *p < 0.05.

Senescent prostate stromal cells promoted cancer cell migration in a paracrine manner. Unconditioned medium (DMEM-CTRL and DMEM-Rg3) and conditioned medium (CM) collected from stromal cells treated with vehicle (CM-CTRL) or ginsenoside Rg3 (CM-Rg3) were used to treat PC3 prostate cancer cells for 24 h in wound-healing assays. CM-CTRL from WPMY-1 significantly promoted the migration of PC3 compared with DMEM-CTRL, while the pro-migratory activity of stromal cells could be reversed by ginsenoside Rg3 (Figure 3A and 3B). Transwell assays were also performed to verify the results (Figure 3C and 3D). Moreover, other wound-healing assays demonstrated that ginsenoside Rg3 also blocked the promoting effects of NAF on cancer cell migration (Supplementary Figure 2).

### Ginsenoside Rg3 inhibited IL-8 expression in prostatic stromal cells

**Figure 4:**
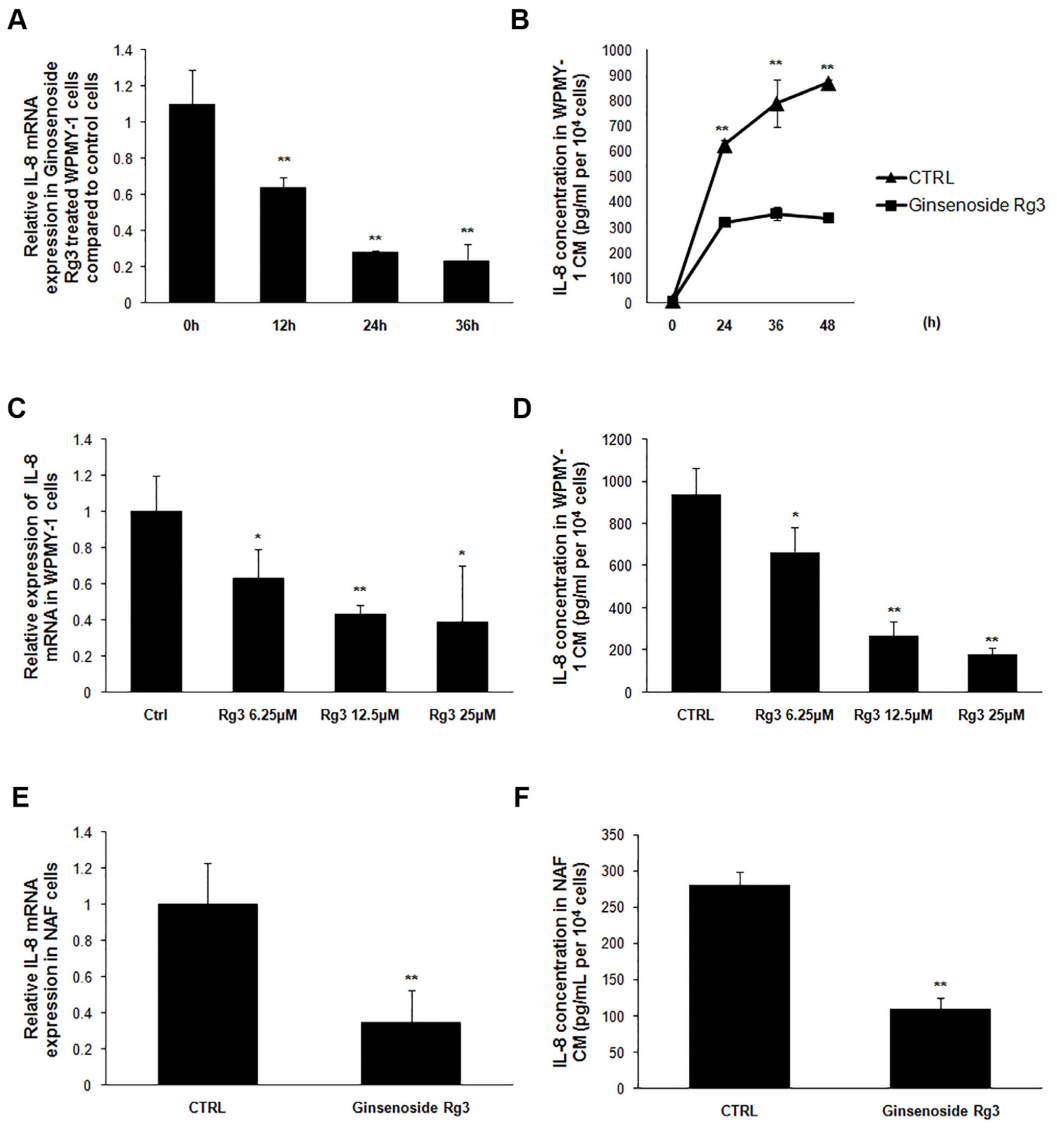
Ginsenoside Rg3 inhibited IL-8 expression in prostate stromal cells **A.** and **B.** show that ginsenoside Rg3 decreased IL-8 expression in a time-dependent manner in WPMY-1 cells. **C.** and **D.** show that ginsenoside Rg3 decreased IL-8 expression in a dose-dependent manner in WPMY-1 cells. **E.** and **F.** show that ginsenoside Rg3 inhibited IL-8 expression in NAF cells. The results were obtained from 3 independent experiments and are presented as means ± SD. *p < 0.05; **p < 0.01.

IL-8 has been reported as up-regulated in senescent fibroblast cells where it contributes to the establishment of a special senescence-associated secretion phenotype (SASP) [13]. In this research, the expression of several factors was detected in stromal cells treated with or without ginsenoside Rg3. IL-8 mRNA expression in WPMY-1 cells and the protein concentration in corresponding CM were significantly down-regulated in a time-dependent manner following treatment with Rg3 (Figure 4A and 4B). Different doses of ginsenoside Rg3 (0, 6.25 μM, 12.5 μM, and 25 μM) were used to treat WPMY-1 cells and dose-dependent down-regulation of IL-8 expression was observed (Figure 4C and 4D). Rg3 also decreased IL-8 expression in NAF cells, both at mRNA and protein level (Figure 4E and 4F).

### Over-expression or exogenous addition of IL-8 blocked the preventive effects of ginsenoside Rg3 on prostate stromal cell senescence

**Figure 5:**
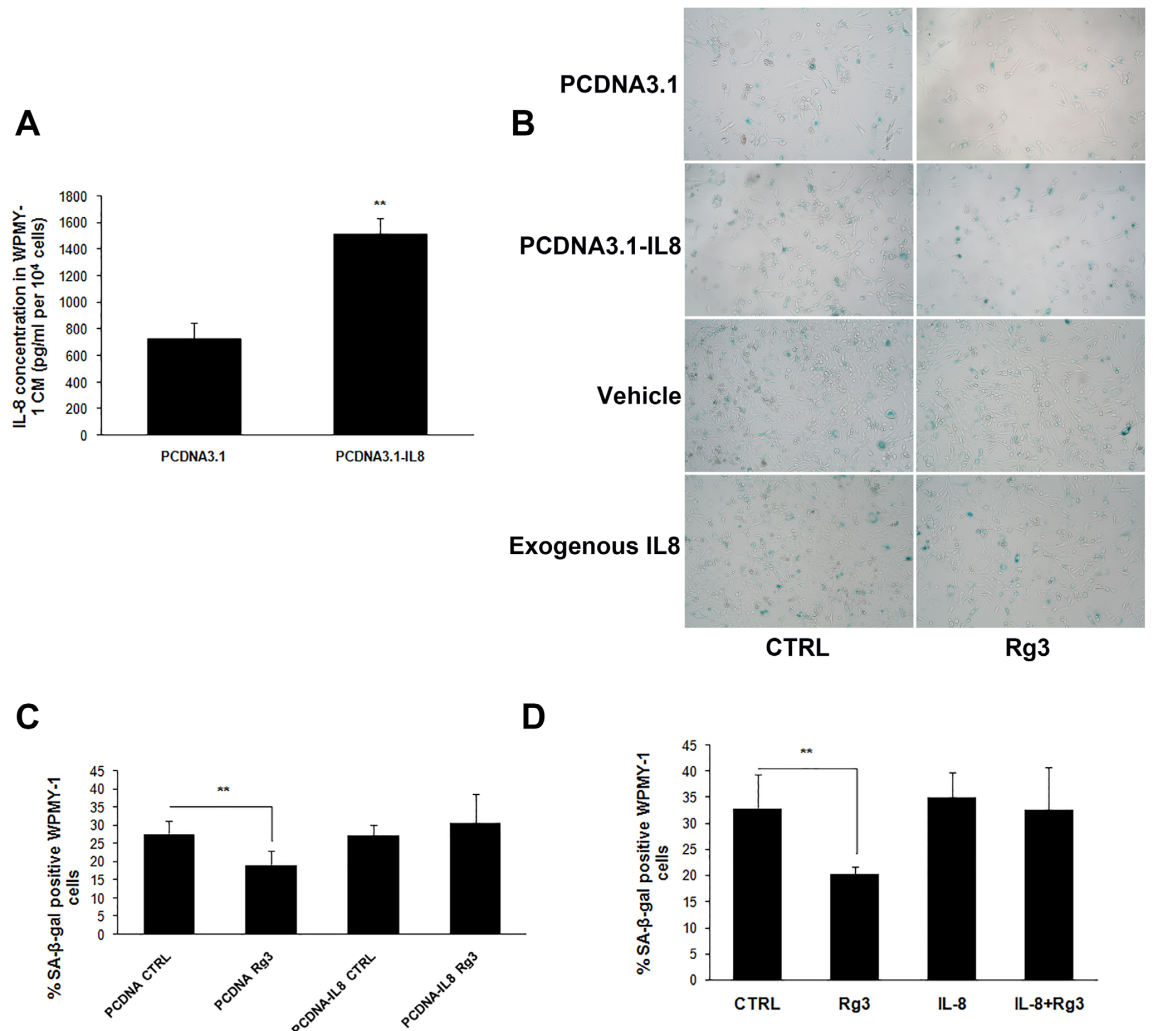
Over-expression or exogenous addition IL-8 blocked ginsenoside Rg3-inhibited prostate stromal cell senescence **A.** Results of ELISA assays confirming the over-expression of IL-8 in WPMY-1 cells transfected with PCDNA3.1-IL8. **B.** shows SA-β-gal staining in WPMY-1 cells transfected with empty vector or PCDNA3.1-IL8 and treated with vehicle or ginsenoside Rg3 as well as SA-β-gal staining in WPMY-1 cells treated with vehicle, Rg3, exogenous IL-8, and Rg3+exogenous IL-8. **C.** and **D.** show the quantitative analysis of the percentage of positive staining cells in (B). The results were obtained from 3 independent experiments and are presented as means ± SD. **p < 0.01.

Previous reports about the role of IL-8 in cellular senescence have been inconsistent. Acosta and Gil [14] discovered that depletion of IL-8 receptor CXCR2 delayed both replicative and oncogenic signal-induced senescence. However, other research has reported that IL-8 may prevent oxidative-stress-induced human endothelial cell senescence [15]. In our study, the plasmid PCDNA3.1-IL8 was constructed and transfected into WPMY-1 cells, and IL-8 over-expression was confirmed by measurement of the protein concentration in CM (Figure 5A). Over-expression of IL-8 reversed ginsenoside Rg3-decreased SA-β-gal positive staining in WPMY-1 cells, while transfection with empty plasmid PCDNA3.1 (+) had no impact on the effects of ginsenoside Rg3. Similarly, exogenous addition of IL-8 to WPMY-1 cells also blocked the anti-senescent role of ginsenoside Rg3 (Figure 5B, 5C, and 5D). Also, the addition of IL-8 antagonized Rg3-inhibited NAF senescence (Supplementary Figure 3). Furthermore, IL-8 addition reversed the damping role of ginsenoside Rg3 in WPMY-1 cell-induced cancer cell migration (Supplementary Figure 4). These results suggested that IL-8 down-regulation was pivotal for ginsenoside Rg3-induced anti-senescence in prostate stromal cells.

### Ginsenoside Rg3 inhibited IL-8 expression by decreasing the cellular ROS level

**Figure 6:**
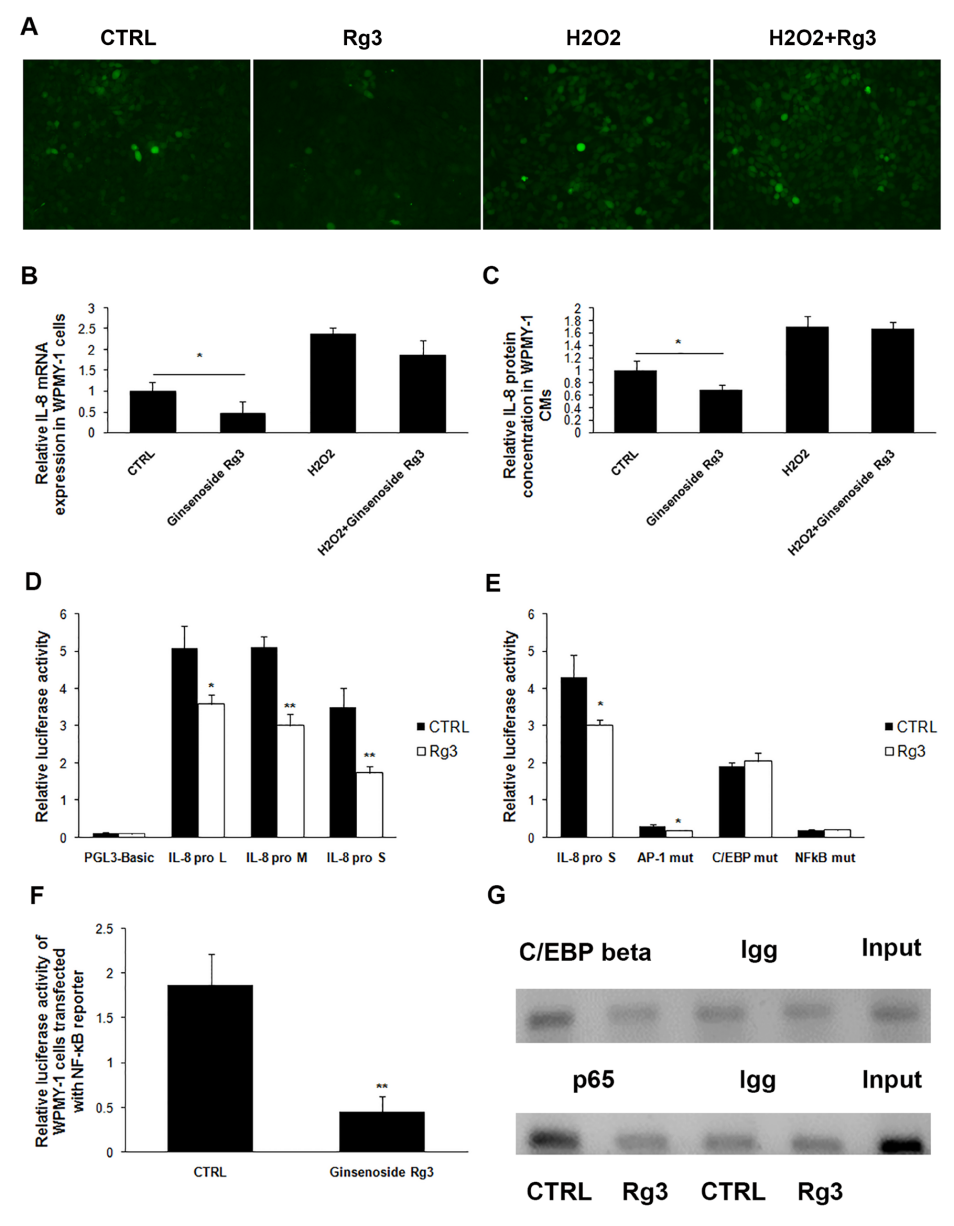
Ginsenoside Rg3 inhibited IL-8 expression in WPMY-1 cells through down-regulation of cellular ROS levels and the transcriptional activity of IL-8 gene promoter **A.** shows images from the use of DCFH-DA to analyze cellular ROS levels in WPMY-1 treated with vehicle, Rg3, H_2_O_2,_ and H_2_O_2_+Rg3. The images were collected at ×200 magnification. **B.** and **C.** show that H_2_O_2_ treatment antagonized the inhibitory role of Rg3 on IL-8 expression. **D.** shows luciferase reporter assay results indicating that ginsenoside Rg3 down-regulated the transcriptional activity of IL-8 promoter. **E.** demonstrates mutation of C/EBP β or NF-κB binding site on IL-8 promoter blocked the inhibitory role of ginsenoside Rg3 on IL-8 promoter transcriptional activity. **F.** Luciferase reporter assay results indicating that ginsenoside Rg3 inhibited NF-κB pathway in WPMY-1 cells. **G.** ChIP assays indicating ginsenoside Rg3 attenuated transcription factor C/EBP β and p65 binding to IL-8 promoter. The results were obtained from 3 independent experiments and are presented as means ± SD. *p < 0.05; **p < 0.01.

The exact molecular mechanism of Rg3 down-regulation of IL-8 expression was further explored. ROS, an indicator of cellular oxidative stress, has been reported to regulate IL-8 expression [16], and ginsenoside Rg3 has been found to suppress LPS- or UV-irradiation-induced ROS levels in macrophage and in the keratinocyte cell line HaCaT [17]. In our study, ROS levels were significantly attenuated in WPMY-1 cells treated with ginsenoside Rg3 for 24 h, while the addition of H_2_O_2_ blocked the regulatory effect of ginsenoside Rg3 on ROS level (Figure 6A). Additionally, IL-8 down-regulation induced by ginsenoside Rg3 was completely antagonized by the addition of H_2_O_2_ (Figure 6B and 6C). CoCl_2_, which was used to induce cell hypoxia, increased ROS in WPMY-1 cells, as well as blocked the regulatory effects of ginsenoside Rg3 on ROS level and IL-8 expression. However, the scavenger of ROS, N-acetyl-L-cysteine (NAC) was able to significantly down-regulate ROS levels and IL-8 protein expression in CoCl_2_-treated WPMY-1 cells (Supplementary Figure 5).

### Ginsenoside Rg3 attenuated the transcriptional activity of IL-8 gene promoter

Three luciferase reporter plasmids, including IL-8 promoter fragments with different lengths - IL-8 pro L (−1481nt to +44nt), IL-8 pro M (−765nt to +44nt), and IL-8 pro S (−176nt to +44nt) - were transfected into WPMY-1 cells and then treated with DMSO or ginsenoside Rg3 for 36 h. The transcriptional activity of all 3 IL-8 promoter fragments was down-regulated by ginsenoside Rg3, suggesting that the regulatory sites responding to ginsenoside Rg3 treatment are located in the shortest fragment (−176nt to +44nt) (Figure 6D). Three main transcription factors AP-1, C/EBP β, and NF-κB are able to bind to this fragment [18]. Each binding site on IL-8 pro S was mutated and the regulatory effects of ginsenoside Rg3 on transcriptional activity were completely blocked when C/EBP β or NF-κB binding site was mutated. Both AP-1 and NF-κB binding site mutation significantly decreased the basic transcriptional activity of IL-8 promoter (Figure 6E). A luciferase reporter plasmid (containing 4 NF-κB conserved motifs) was transfected into WPMY-1 cells, and ginsenoside Rg3 down-regulated both the basic and TNFα-enhanced luciferase activity significantly (Figure 6F and Supplementary Figure 6). These results demonstrated that Rg3 inhibited NF-κB signaling pathway activation in WPMY-1 cells. Moreover, chromatin immunoprecipitation (ChIP) assays demonstrated that ginsenoside Rg3 attenuated C/EBP β and p65 binding to the corresponding sites (Figure 6G). Another ChIP assay indicated that H_2_O_2_ addition blocked the regulatory effects of ginsenoside Rg3 on p65 binding to IL-8 promoter (Supplementary Figure 6B).

## DISCUSSION

The accumulation of senescent cells has been demonstrated during organism aging [19], which suggests that senescent cells may play a role in aging-associated disease, including prostate cancer. Cellular senescence seems to exhibit diverse effects on epithelial and stromal cells in prostate cancer [20, 21]. Senescence is considered to prevent normal prostate epithelial cells from neoplasia as epithelial senescence exists in BPH and prostate intraepithelial neoplasia but is rare in frank tumors [22]. Besides, elevated expression of the senescent marker β-galactosidase is associated with lower cancer stage and predicts reduced prostate specific antigen (PSA) recurrence within primary prostate tumor [23]. Although senescence in prostate epithelial cells is considered to be anti-tumorigenic, senescent stroma promotes cancer progression through the secretion of chemokines/cytokines and the remolding of extracellular matrix, providing a pro-tumorigenic microenvironment.

Ginsenoside Rg3 has been reported to inhibit prostate cancer cell proliferation and migration, and enhance cancer cell sensitivity to chemotherapy. However, the effects of ginsenoside Rg3 on prostate stromal cells have not been clarified. Our research revealed the anti-senescent effects of ginsenoside Rg3 through a decrease in SA-β-gal positive staining in prostatic stromal cells, both by incubation in low serum medium and by over-replication. Under serum starvation conditions, ginsenoside Rg3 promoted cell cycle transition to S phase from G0/G1 during which senescent cells were arrested [24], down-regulated p21 expression (Supplementary Figure 1), increased cell viability and proliferation, and inhibited stromal cell-induced cancer cell migration. In a replicative senescent model of primary cultured rat prostatic stromal cells, ginsenoside Rg3 decreased p21 expression and DNA damage, as indicated by the positive staining of γH2A.X that was observed in the replicative senescent stromal cells but not in cells treated with ginsenoside Rg3. Furthermore, no γH2A.X staining was observed in stromal cells incubated in serum starvation conditions.

SM22 is a 22KD actin-binding protein mainly expressed in smooth muscle cells and is considered a marker of smooth muscle differentiation. SM22 can act as a cancer repressor by modulating the secretion of matrix metalloproteinase 9 (MMP9) or the AR signaling pathway [25, 26]. Another smooth muscle marker, SMMHC, is also reported to be down-regulated either in BPH or smooth muscle adjacent to prostate cancer sites [27, 28]. These results provide some clues about the phenotype of pro-carcinogenic stromal cells in the prostate. Our recent report also demonstrated the down-regulation of SM22 and SMMHC in carcinoma associated stromal cells [29]. Although the exact differentiation phenotype of senescent prostate stromal cells has not been fully identified, this research revealed an up-regulation of SM22 and SMMHC in ginsenoside Rg3-treated cells, indicating the compound may inhibit the pro-carcinogenic stromal phenotype.

Inflammatory factors, such as IL-6 and IL-8, contribute to cellular growth arrest and senescence [30, 31]. Our research evaluated the expression of several factors in ginsenoside Rg3-treated WPMY-1 cells and only down-regulation of IL-8 was observed (Supplementary Figure 7). The role of IL-8 in cellular senescence is controversial. Medina et al. [32] pointed out that IL-8 is a facilitator of the outgrowth of endothelial cell senescence, while Shen et al. [15] highlighted the preventive role of IL-8 on oxidative stress-induced endothelial cell senescence. In our study, over-expression or exogenous addition of IL-8 completely reversed the inhibitory effects of ginsenoside Rg3 on the senescence of stromal cells incubated in low serum medium and on cancer cell migration induced by CM collected from stromal cells. These results identified IL-8 as the pivotal modulator in ginsenoside Rg3-prevented stromal cell senescence.

IL-8 has been reported as a potential therapeutic target in prostate cancer treatment [33], while the regulatory model of IL-8 expression in prostate stroma has not been fully elucidated. Previous report has suggested that ROS and the NF-κB pathway are involved in IL-8 regulation [34]. In this research, ginsenoside Rg3 decreased ROS in stromal cells incubated in serum starvation conditions, while either H_2_O_2_ or CoCl_2_ up-regulated ROS levels and blocked the inhibitory effects of ginsenoside Rg3 on both ROS level and IL-8 expression. It was interesting that the ROS scavenger NAC, but not ginsenoside Rg3, was able to attenuate CoCl_2_-induced ROS increase and IL-8 expression. First, the results suggested that ROS has a regulatory role in IL-8 expression, as depletion of ROS by NAC down-regulated IL-8 expression. Second, ginsenoside Rg3 and H_2_O_2_/CoCl_2_ may have different regulatory mechanisms in ROS modulation. Cellular ROS is mainly generated by mitochondria and NADPH oxidases (NOXs). We found that Rg3 down-regulated the mRNA expression of NOX4 (Supplementary Figure 7), which is one producer of ROS in cells [35], while a recent research indicated that depleting of mitochondrial DNA in liver tumor cells impaired CoCl_2_ induced ROS generation, which suggested mitochondria with intact function were essential in CoCl_2_ induced ROS up-regulation [36]. However, the exact role of NOX4 in the ginsenoside Rg3 regulation of IL-8 expression and in cellular senescence needs further exploration. Our research also analyzed transcriptional activity of the IL-8 gene promoter and revealed ginsenoside Rg3 decreased the recruitment of C/EBP β and p65 to their corresponding binding sites on the IL-8 promoter.

In conclusion, our study demonstrated that ginsenoside Rg3 prevented the senescence in prostate stromal cells, and inhibited cancer cell migration through a stromal-epithelial interaction. Ginsenoside Rg3 also decreased the IL-8 expression essential for inhibiting serum-starved stromal cell senescence. Decreased cellular ROS and attenuation of the binding of transcription factors C/EBP β and p65 to IL-8 promoter mediated the inhibitory effects of ginsenoside Rg3 on IL-8 expression. Recent reports indicate that chemotherapy may induce stromal cell senescence, contributing to the chemoresistance of cancer cells [37] and this may be a possible reason for clinical chemotherapy failure. The research results lead to the novel suggestion of targeting the senescent stromal microenvironment, and assist in objective assessment of the use of ginsenoside Rg3 in prostate cancer treatment.

## MATERIALS AND METHODS

### Cell culture and treatment

Normal prostate stromal cell line WPMY-1 (American Type Culture Collection, USA) was cultured in DMEM medium (Sigma, USA), supplemented with 5% FBS (Invitrogen, USA) and 1% penicillin/streptomycin (P/S, Hyclone, USA). NAF (Normal-associated fibroblast) and CAF (carcinoma-associated fibroblast) cells, supplied by the Department of Urology of Medical University of Innsbruck (Innsbruck, Austria), were cultured in DMEM medium supplemented with 10% FBS and 1% P/S. Prostate cancer cells PC3, supplied from the German Cancer Research Center (DKFZ) (Germany), were cultured in RPMI 1640 medium (Sigma, USA) supplemented with 1% P/S and 10% FBS. WPMY-1, NAF, and CAF cells were pre-incubated in DMEM medium supplemented with 0.5% FBS for 36 h followed by treatment with either vehicle (DMSO) or 25 μM ginsenoside Rg3 (YiFang S&T, China).

### SA-β-gal staining

WPMY-1, NAF, and CAF cells were seeded into 6-well plates and treated with vehicle or ginsenoside Rg3 for 48 h. A commercial SA-β-gal staining kit (Beyotime Institute of Biotechnology, China) was used according to the manufacturer^’^s instructions. Senescent cells were identified as blue-stained by standard light microscopy (Leica, Germany), and photographs were taken using a phase-contrast microscope. Six view fields were randomly selected and the percentage of positive stained cells was calculated. The results were expressed as mean ± standard deviation (SD).

### Cell viability assays

WPMY-1 and NAF cells were treated with either vehicle or 25 μM ginsenoside Rg3 for 0, 24, and 48 h, respectively. 3-(4, 5-Dimethylthiazol-2-yl)-2, 5-diphenyltetrazolium bromide (MTT) (Sigma, USA) was added into the medium. Cell medium was discarded 2 h later and 250 μL DMSO was added into each well. The absorbance at 570 nm was measured with a microplate reader (BioTek, USA). The results were expressed as mean of triplicates ± SD.

### Cell cycle analysis

WPMY-1 and CAF cells were treated with vehicle or ginsenoside Rg3 for 24 h. Flow cytometry assays were used to analyze the cell cycle as previously reported [38].

### Immunofluorescence assays

WPMY-1 and NAF cells were cultured onto the slides and treated with vehicle or ginsenoside Rg3 for 72 h. The slides were then fixed with 4% paraformaldehyde, washed with phosphate buffered saline (PBS) and treated with PBS containing 10% FBS to block nonspecific reactions. The slides were incubated with primary antibodies (rabbit anti-SM22 [sc-50446, Santa Cruz, USA] and mouse anti-SMMHC [sc-6956, Santa Cruz, USA]) overnight at 2-8 °C. Secondary antibodies (Alexa Fluor 488 donkey anti-rabbit IgG [H+G] and Alex Fluor 594 goat anti-mouse IgG [H+L] [Life Technologies, USA]) labeled with green/red fluorescence were used for 1 h at 37 °C. The cell nucleus was labeled with 4, 6-diamino-2-phenyl indole (DAPI) (Sigma, USA). Images were taken using a fluorescent microscope (Leica, Germany) at 400 × magnification.

### Western blot

WPMY-1 and NAF cells were treated with vehicle or ginsenoside Rg3 for 72 h. Proteins of cell extracts were resolved by sodium dodecyl sulfate polyacrylamide gel electrophoresis and transferred onto polyvinylidene difluoride (PVDF) membranes. The membranes were incubated with antibodies against SM22, SMMHC, and GAPDH (KC-5G4, Kangchen, China) overnight at 2 -8 °C. Proteins were detected by appropriate secondary antibodies (goat anti-rabbit or goat anti-mouse) conjugated with horseradish peroxidase (Bio-Rad, USA) followed by enhanced chemiluminescence detection (Amersham, USA). The results were quantified with Image J software and expressed as mean of triplicates ± SD.

### Wound-healing assays

WPMY-1 cells were treated with vehicle or ginsenoside Rg3 for 48 h. CMs were collected, centrifuged at 400 g for 5 min to remove the floating cells and normalized according to cell number and named CM-CTRL and CM-Rg3 respectively. Unconditioned mediums (unCMs) DMEM-CTRL and DMEM-Rg3 (incubated DMEM medium supplied with vehicle or 25 μM Rg3 in a cell incubator for 48 h) were used as control. PC3 cells were cultured with RPMI1640 medium containing 0.5% FBS for 24 h. Each well was scratched with disposable tips in the cell monolayer. Each CM or unCM was mixed with RPMI1640 supplemented with 0.5% FBS in a 1:2 ratio. PC3 cells were treated with each mixture for 24 h. Images were taken at 200 × magnification under a phase-contrast microscope (Leica, Germany). Image J software was used to analyze the relative migration distance. The results were expressed as mean of triplicates ± SD.

### Transwell assays

A protocol for transwell assays was followed according to our previous report [29]. Results were obtained from 3 independent experiments and quantitative results expressed as mean ± SD.

### RNA isolation and real time PCR

Total RNA was extracted using Trizol reagent (Invitrogen, USA). Reverse transcription was performed with a commercial reverse transcription kit (Promega, USA). Real-time polymerase chain reaction (PCR) was performed using the specific primers listed in Table 1. The relative gene expression was determined using the comparative CT method and normalized to housekeeping gene hypoxanthine phosphoribosyltransferase 1 (HPRT).

**Table 1:** Nucleotide sequence of primers used in this research

Primer name	Sequence (5, - 3,)	Purpose
Human IL8 Sa	CTCTGGCAGCCTTCCTGATT	Real-time PCR
Human IL8 ASb	TATGCACTGACATCTAAGTTCTTTAGC	Real-time PCR
Human HPRT S	TGACACTGGCAAAACAATGCA	Real-time PCR
Human HPRT AS	GGTCCTTTTCACCAGCAAGCT	Real-time PCR
IL8 clone S	ATAGGATCCCCGGAAGGAACCATCTCA	PCDNA3.1-IL8
IL8 clone AS	AATCTCGAGCTGGCATCTTCACTGATTCTTG	PCDNA3.1-IL8
IL8 promoter-1481	CGAGGTACCGAATTCAGTAACCCAGGCATTATT	IL-8 pro L
IL8 promoter-765	CGAGGTACCGCTCTTATGCCTCCACTG	IL-8 pro M
IL8 promoter-176	CGAGGTACCAAAACTTTCGTCATACTCCG	IL-8 pro S
IL8 promoter+44	ATAAGATCTAGCTTGTGTGCTCTGCTGTCTCTGAAA	IL-8 pro L, M, S
IL8 AP1 S	TGTGATATCTCAGGTTTGCCCTG	AP-1 mut
IL8 AP1 AS	CAAACCTGAGATATCACACTTCCT	AP-1 mut
IL8 C/EBP S	GCCATAGCTTGCAAATCGTGGAAT	C/EBP mut
IL8 C/EBP AS	TTTGCAAGCTATGGCCCATCCC	C/EBP mut
IL8 NFκB S	ATCGTTAACTTTCCTCTGACATA	NF-κB mut
IL8 NFκB AS	AAAGTTAACGATTTGCAACTGATG	NF-κB mut
ChIP S	ACTTTCGTCATACTCCG	ChIP assay
ChIP AS	CACCCTCATCTTTTCAT	ChIP assay

a, Sense; b, Antisense.

### Human IL-8 ELISA assays

CM was collected from WPMY-1 or NAF cells and centrifuged at 400 g for 5 min to remove the floating cells. CM volumes were normalized to cell numbers. The concentration of IL-8 in CM was determined with ELISA kits (Boster, China) following the protocol of the manufacturer.

### Plasmids and transfection

Human IL-8 cDNA fragment containing complete coding DNA sequence (CDS) region was obtained from the total RNA of WPMY-1 cells by reverse transcription and PCR amplification. The cDNA fragment was cloned into vector PCDNA3.1 (+) with the restriction enzymes BamH I and Xho I (Takara, Japan) and the over-expression plasmid was named PCDNA3.1-IL8. Human IL-8 gene promoter fragment (−1481nt to +44nt) was amplified from genomic DNA isolated from WPMY-1. The fragment was cloned into PGL3-Basic plasmid with Kpn I and Bgl II (Takara, Japan) to construct the luciferase reporter plasmid IL-8 pro L (−1481nt to +44nt). Another two sense primers were used to construct the other two luciferase reporter plasmids: IL-8 pro M (−765nt to +44nt) and IL-8 pro S (−176nt to +44nt). The primers are listed in Table 1. The plasmid pNF-κB-luc was a luciferase reporter with 4 NF-κB motifs (Clontech, USA). Transfections were performed using Lipofectamine™2000 (Invitrogen, USA). Luciferase assays were performed as described previously [39].

### Exogenous IL-8 addition

WPMY-1/NAF cells were treated with vehicle, 25 μM ginsenoside Rg3, 10 ng/mL human IL-8 protein (GenScript, USA), and human IL-8 protein + Rg3. After 48 h, the cells were analyzed with SA-β-gal staining and the percentage of positive stained cells was calculated. The results were expressed as mean ± SD.

### ROS level assays

WPMY-1 cells were treated with DMSO, 25 μM Rg3, 600mM H_2_O_2_ (Solarbio, China) and Rg3+H_2_O_2_, respectively. After 24 h, an ROS assay kit (Beyotime institute of biotechnology, China) was used to evaluate ROS levels according to the protocol. The photographs were taken under a fluorescent microscope (Leica, Germany) at 200 × magnification.

### *In vitro* mutagenesis of the AP-1, C/EBP β, and NF-κB binding sites

The AP-1, C/EBP β, and NF-κB binding sites on IL-8 promoter were mutated by PCR respectively. The luciferase reporter plasmid IL-8 pro S was used as the template and the primers are listed in Table 1. The PCR products were digested by Dpn I (Takara, Japan) for 3 h and transferred into DH5α bacteria to obtain 3 corresponding mutated reporter plasmids, named as AP-1 mut, C/EBP mut, and NF-κB mut. Each mutagenesis was confirmed by DNA sequencing.

### ChIP assays

WPMY-1 cells were treated with vehicle or ginsenoside Rg3 for another 24 h. ChIP assays were then performed as described previously [39]. C/EBP β (sc-150, Santa Cruz, USA) and p65 antibodies (ab7970, Abcam, UK) and control rabbit Igg (bs-0296P, Bioss, China) were used. The primers used in the ChIP assays are shown in Table 1 and the PCR product was 128 bp.

### Statistical analysis

The results are expressed as mean ± SD. Significance was assessed using Student’s paired t-test. *P* < 0.05 was considered significant and *p* < 0.01 highly significant.

## SUPPLEMENTARY MATERIALS FIGURES


