# Supplementary material for: Ginsenoside Rg3 inhibits the senescence of prostate stromal cells through down-regulation of interleukin 8 expression
Source: Oncotarget. 2017 May 4;8(39):64779–92. doi: 10.18632/oncotarget.17616 (PMC5630291; doi:10.18632/oncotarget.17616)
Supplement: Supplementary file 1 [file oncotarget-08-64779-s001.pdf]

Ginsenoside Rg3 inhibits the senescence of prostate stromal cells through down-regulation of interleukin 8 expression

Supplementary Material

Supplementary Materials and Methods

Cells and materials

The primary-cultured rat prostate stromal cells (donated by Department of Biochemistry and Molecular Biology, Nankai University, China) were cultured in DMEM medium supplemented with 10% FBS and 1% P/S. Anti-p21 antibody (Bioss, China) was used to evaluate p21 expression by Western blot and immunofluorescence assays. Anti-γH2A.X antibody (Abcam, UK) was used to detect γH2A.X foci in stromal cells by immunofluorescence assays. NAC (Beyotime Institute of Biotechnology, China) was used to scavenge ROS in $CoCl_2$-treated WPMY-1 cells.

Replicative senescence of primary-cultured cells

Third-generation primary-cultured rat stromal cells were treated with vehicle or 25 µM ginsenoside Rg3. The 2 groups of cells were cultured until eighth generation and the cells were fixed and SA-β-gal stained. Immunofluorescence assays were also performed to detect γH2A.X foci and p21 expression.

CoCl2 treatment

WPMY-1 cells were treated with vehicle, 400 µM $CoCl_2$, 500 µM NAC+$CoCl_2$, or 25 µM ginsenoside Rg3+$CoCl_2$. The cells were pre-treated with NAC or ginsenoside Rg3 for 1 h before $CoCl_2$ addition. ROS levels were detected after 24 h. IL-8 protein expression was analyzed with ELISA assay after 48 h.

TNFα treatment

WPMY-1 cells were transfected with pNF-κB-luc and then treated with vehicle, 20ng/mL TNFα, or 25 µM ginsenoside Rg3+TNFα. TNFα was added into medium 1 h before ginsenoside Rg3 treatment. The relative luciferase activity was detected after 48 h.

Animals and treatment

Thirteen male balb/c mice (6 weeks of age) were purchased from Tianyao Inc (Tianjin, China) and acclimated in an animal-care facility for 1 week. The mice were randomly divided into 3 groups: control group (normal saline was daily intraperitoneal injected for 6 weeks, n=5), D-gal group (200mg/kg D-gal was daily intraperitoneal injected for 6 weeks and oral gavage of saline was daily performed from the 10th day, n=4) and D-gal+Rg3 group (200mg/kg D-gal was daily intraperitoneal injected for 6 weeks and oral gavage of 20mg/kg ginsenoside Rg3 was daily performed from the 10th day, n=4). After 6 weeks, the mice were executed and the prostates were isolated and made into frozen sections. Then SA-β-gal staining assays were performed and all the photographs were taken using a phase-contrast microscope at 200× magnification. At the same time, the serum samples of mice were also collected and CXCL1 ELISA kit (Boster, China) and GROb ELISA kit (USCN KIT INC, China) were used to detect serum CXCL1 and GROb levels. All experiments were approved by the Animal Experimentation Ethics Committee of Tianjin International Joint Academy of Biotechnology and followed the institute guidelines.

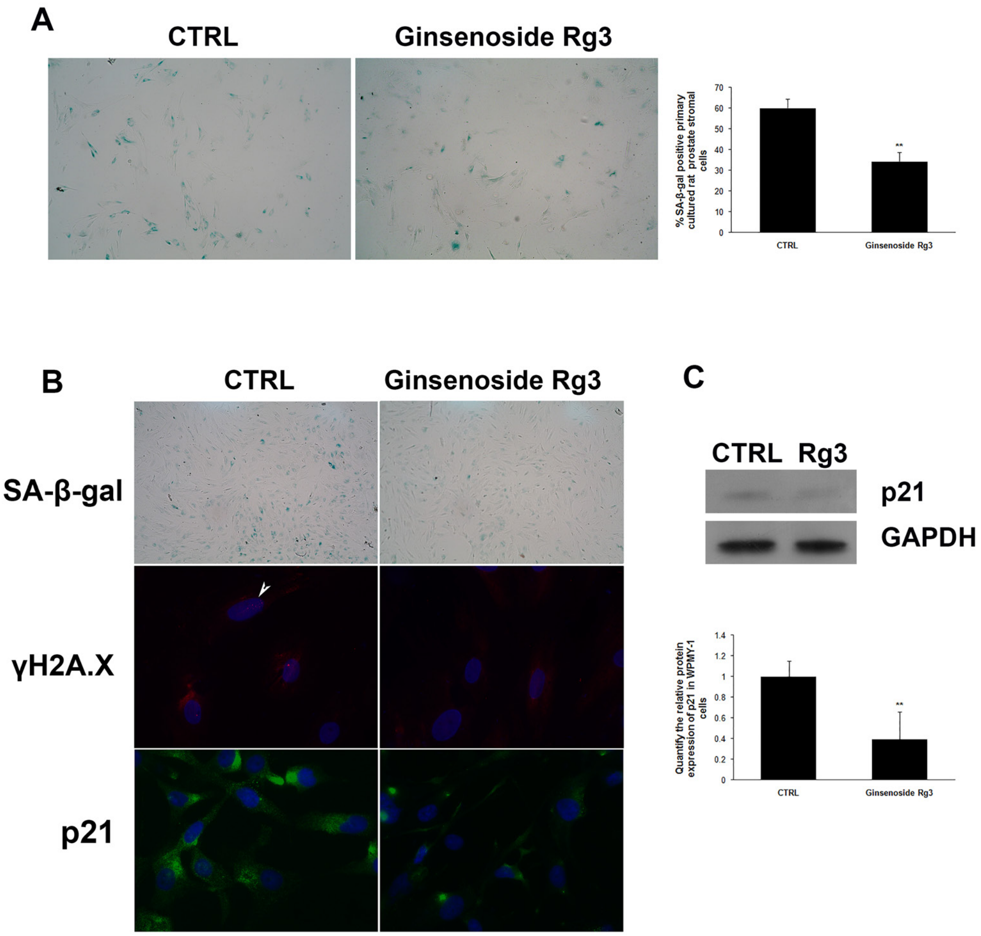

Supplementary Figure S1: Ginsenoside Rg3 inhibited serum-starvation induced and replicative senescence of primary cultured rat prostate stromal cells. (A) Images showing that ginsenoside Rg3 decreased SA-β-gal positive staining in primary cultured cells incubated in serum starvation conditions. The photographs were taken at ×100 magnification. (B) Ginsenoside Rg3 decreased SA-β-gal positive staining (×100) and γH2A.X staining (×400, white arrow indicating), as well as p21 expression (×400) in primary cultured cells at the eighth generation. (C) Ginsenoside Rg3 down-regulated p21 expression in WPMY-1 cells incubated in serum starvation conditions. The results were obtained from 3 independent experiments and are presented as means ± SD. **$p < 0.01$.

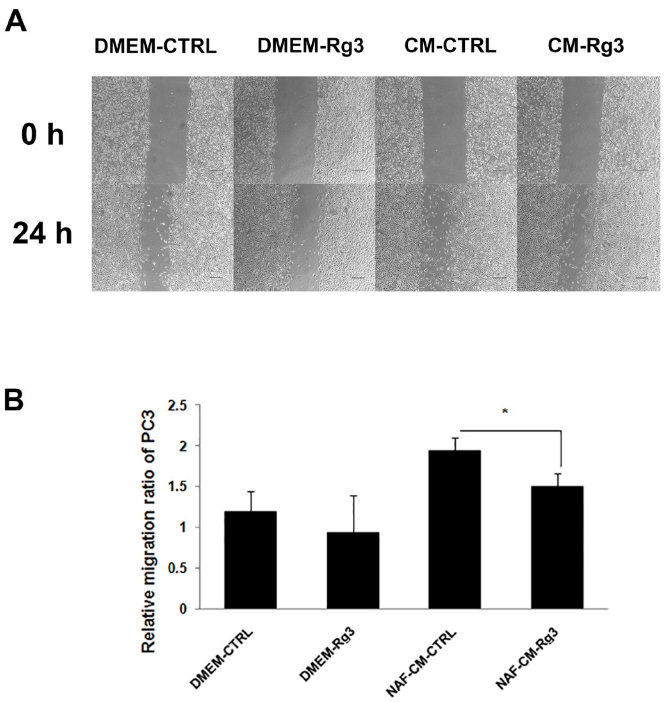

Supplementary Figure S2: Wound healing assays indicated ginsenoside Rg3 inhibited PC3 cell migration by modulating NAF cell paracrine. (A) Wound healing assays of PC3 cells at 0 and 24 h. The pictures were captured at ×40 magnification. Scale bar, 500 µm. (B) Quantification of the results from wound healing assays. The results were obtained from 3 independent experiments and are presented as means ± SD. *p < 0.05.

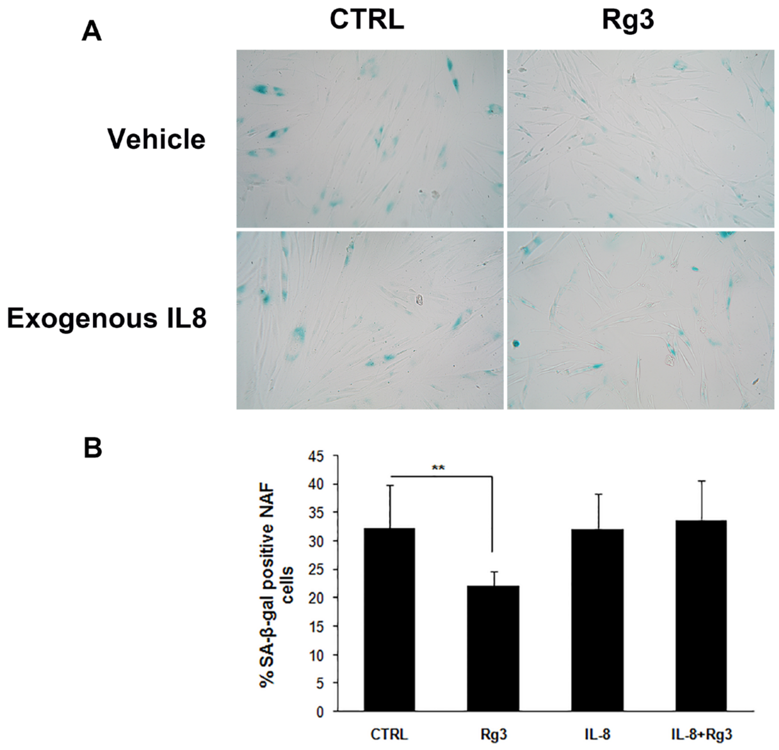

Supplementary Figure S3: The addition of exogenous IL-8 blocked ginsenoside Rg3-inhibited NAF cell senescence. (A) The images show SA-β-gal staining in NAF cells treated with vehicle, Rg3, exogenous IL-8, and Rg3+exogenous IL-8, captured at ×100 magnification. (B) The quantitative results were obtained from 3 independent experiments and are presented as means ± SD. **p < 0.01.

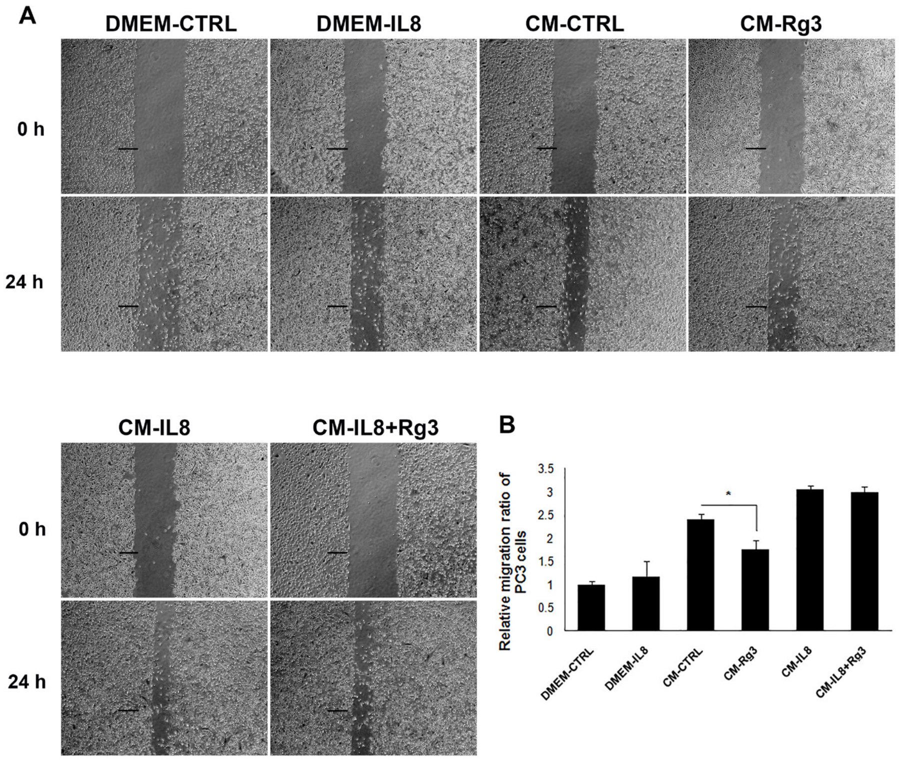

Supplementary Figure S4: The addition of exogenous IL-8 blocked the inhibitory effects of ginsenoside Rg3 on WPMY-1 cell-induced cancer cell migration. (A) Wound healing assays of PC3 cells at 0 and 24 h are shown. The images were collected at ×40 magnification. Scale bar, 500 µm. (B) The quantitative results were obtained from 3 independent experiments and are presented as means ± SD. $^*p < 0.05$.

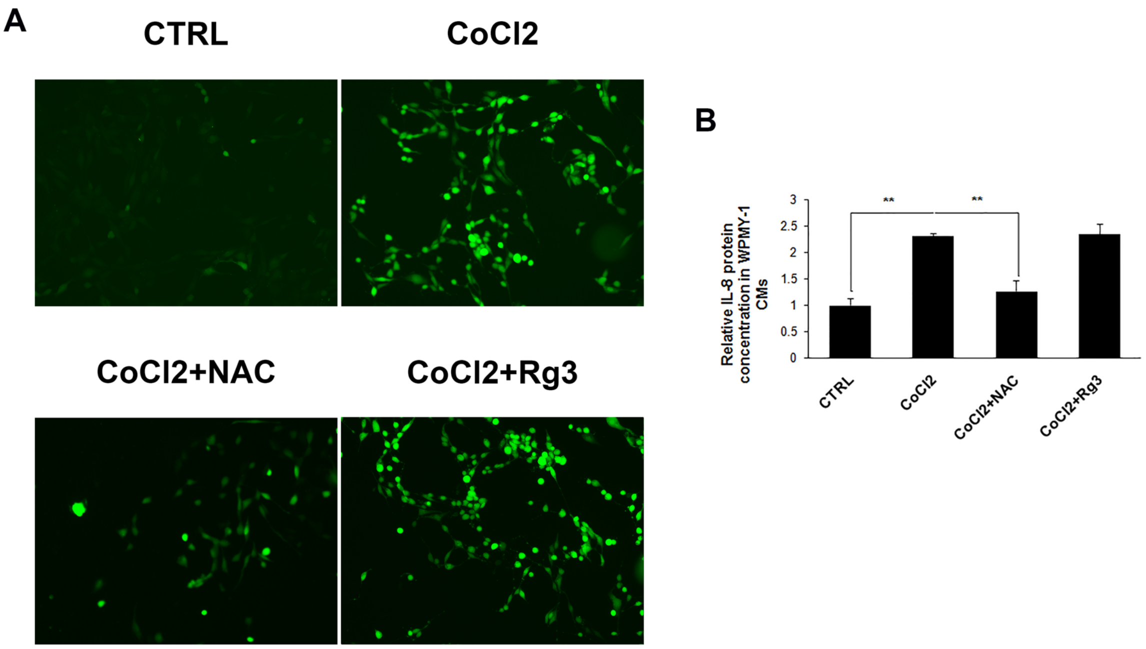

Supplementary Figure S5: $CoCl_2$ treatment antagonized ginsenoside Rg3-decreased ROS and IL-8 expression in WPMY-1 cells. (A) shows the results of analysis using DCFH-DA of cellular ROS levels in WPMY-1 treated with vehicle, Rg3, $CoCl_2$, $CoCl_2$+NAC, and $CoCl_2$+Rg3. The images were collected at ×100 magnification. (B) ELISA assay results indicating that $CoCl_2$ treatment blocked the down-regulation of IL-8 protein expression induced by ginsenoside Rg3. The results were obtained from 3 independent experiments and are presented as means ± SD. $^{**}p < 0.01$.

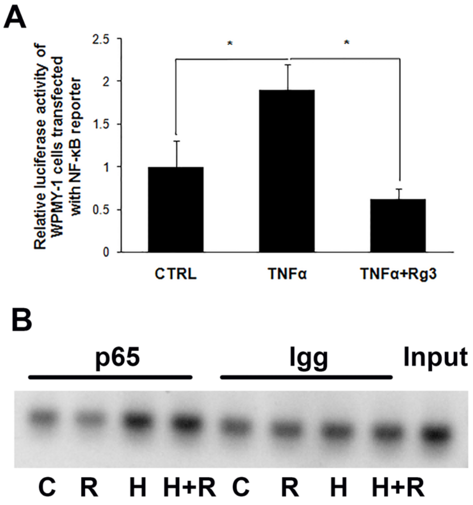

Supplementary Figure S6: Ginsenoside Rg3 inhibited the NF-κB pathway in WPMY-1 cells. (A) Results for luciferase reporter indicating ginsenoside Rg3 down-regulated TNFα-induced NF-κB activation. (B) ChIP assays suggesting H_2O_2 addition blocked the inhibitory effects of ginsenoside Rg3 on recruiting p65 to IL-8 promoter. C: control; R: ginsenoside Rg3; H: H_2O_2; H+R: H_2O_2+ ginsenoside Rg3.

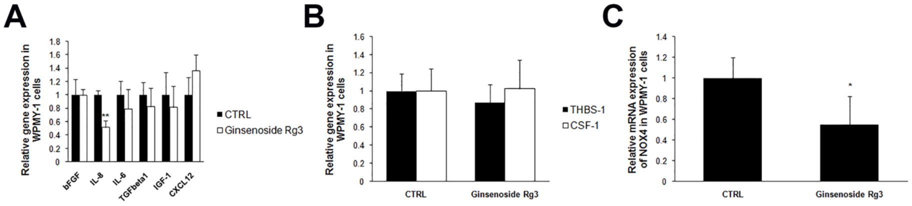

Supplementary Figure S7: The expression of several genes was detected in WPMY-1 cells treated with ginsenoside Rg3. (A) Real-time RT-PCR results indicating that cytokines bFGF, IL-6, TGFβ1, IGF-1 and CXCL12 were not regulated in WPMY-1 cells treated with ginsenoside Rg3. (B) Real-time RT-PCR indicating that THBS1 and CSF1 were not regulated in WPMY-1 cells treated with ginsenoside Rg3. (C) Real-time RT-PCR indicating ginsenoside Rg3 decreased NOX4 expression in WPMY-1 cells. All the primers are listed in Table S1.The results were obtained from 3 independent experiments and are presented as means ± SD. *$p < 0.05$; **$p < 0.01$.

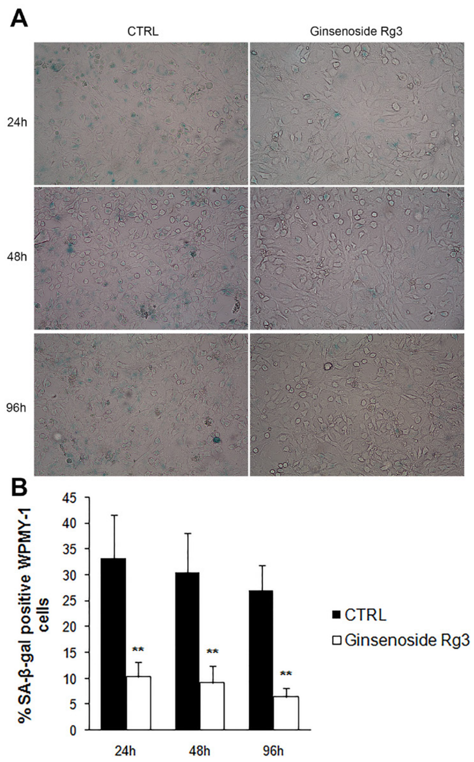

Supplementary Figure S8: Ginsenoside Rg3 down-regulated the positive SA-β-gal staining in serum-starved WPMY-1 cells at 24, 48 and 96h. (A) Images showing SA-β-gal staining in WPMY-1 cells were collected at ×400 magnification. (B) Quantitative analysis of the percentage of SA-β-gal positive stained cells. The results were obtained from 3 independent experiments and are presented as means ± SD. $^{**}p < 0.01$.

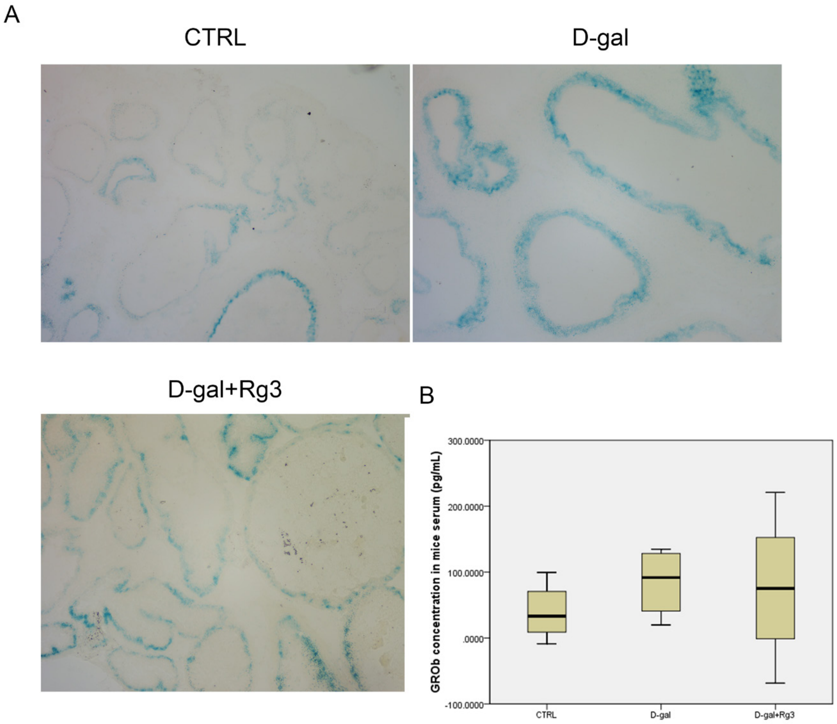

Supplementary Figure S9: Ginsenoside Rg3 decreased positive SA-β-gal stained prostatic cells in D-galactose induced aging mice. (A) The frozen sections of the prostates collected from mice were used in SA-β-gal staining assays. The photographs were taken at 200× magnification. (B) ELISA analysis of the serum GROb levels. Ctrl group, n=5. D-gal group, n=4. D-gal+Rg3 group, n=4.
